# Better Together: acceptability, feasibility and preliminary impact of chronic illness peer support groups for South African adolescents and young adults

**DOI:** 10.1002/jia2.26148

**Published:** 2023-11-01

**Authors:** Abigail Harrison, Bulelwa Mtukushe, Caroline Kuo, Marta Wilson‐Barthes, Bianca Davidson, Rebecca Sher, Omar Galárraga, Jacqueline Hoare

**Affiliations:** ^1^ International Health Institute Brown University School of Public Health Providence Rhode Island USA; ^2^ School of Public Health and Family Medicine University of Cape Town Cape Town South Africa; ^3^ Division of Psychiatry & Groote Schuur Hospital University of Cape Town Cape Town South Africa; ^4^ Department of Health Studies American University Washington DC USA

**Keywords:** adolescents, HIV care continuum, social support, Africa, coinfection, stigma

## Abstract

**Introduction:**

Peer support can help navigate the isolation and psychological strain frequently experienced by youth living with chronic illness. Yet, data are lacking on the impact of providing support for youth living with mixed chronic conditions. We assessed the acceptability, feasibility and preliminary mental health impacts of a clinic‐based peer support group for South African youth living with chronic illnesses, including HIV.

**Methods:**

This mixed‐methods pilot study (September 2021–June 2022) enrolled 58 young patients, ages 13–24, at an urban hospital in Cape Town, South Africa. In‐depth interviews elicited the perspectives of 20 young people in relation to their participation in the *Better Together* programme, a recurring clinic‐based peer support group for patients with mixed chronic illnesses. Self‐reported resilience, attitudes towards illness, stigma and mental health were captured via established measures. *T*‐tests and multivariate analysis of variance compared psychosocial outcomes for 20 group participants and 38 control patients, controlling for socio‐demographic characteristics at enrolment. Logistic regression analyses estimated the predicted probability of a positive depression or anxiety screening given peer group participation.

**Results:**

All interviewees valued being able to compare treatment regimens and disease management habits with peers living with different conditions. Adolescents living with HIV stated that understanding the hardships faced by those with other conditions helped them accept their own illness and lessened feelings of isolation. Compared to patients who did not participate in *Better Together*, those who attended ≥5 groups had statistically significantly higher individual‐level resilience, a more positive attitude towards their illness(es), lower internalised stigma and a more positive self‐concept. The probability of being screened positive for depression was 23.4 percentage points lower (95% CI: 1.5, 45.3) for *Better Together* participants compared to controls; the probability of a positive anxiety screening was 45.8 percentage points lower (95% CI: 18.1, 73.6).

**Conclusions:**

Recurring, clinic‐based peer support groups that integrate youth living with HIV and other chronic diseases are novel. Group sustainability will depend on the commitment of experienced peer leaders and providers, routine scheduling and transportation support. A fully powered randomised trial is needed to test the optimal implementation and causal mental health effects of the *Better Together* model.

## 1 INTRODUCTION

Sub‐Saharan Africa (SSA) has some of the worst health profiles for young people in the world [1]. Ninety percent of the 2.1 million young people living with HIV reside in SSA [2, 3], and AIDS remains the leading cause of death among young people in the region [4]. Chronic kidney disease is increasing among young people in SSA [5, 6, 7] and the annual incidence of type 1 diabetes is 3.9% in this population [8]. Chronic illness, including HIV, is a leading risk factor for depression and other psychological disorders in youth [5, 8, 9, 10, 11], with young people with chronic conditions feeling isolated and misunderstood by their healthy peers [12, 13, 14, 15]. As demonstrated in the HIV and AIDS literature, feelings of social isolation are further compounded by reduced access to care [16, 17] and disease‐related stigma [18, 19], which can undermine treatment adherence [5, 12, 16, 20, 21, 22, 23, 24] and contribute to poorer outcomes [12, 14, 16, 25, 26, 27, 28].

Adolescence is a time of rapid growth and physiological change, accompanied by important individuation and socialisation processes [29]. Managing a chronic disease during this developmental stage often presents challenges for the patient as well as their family and care providers; medication adherence and retention in chronic disease care are frequently worse for adolescents than for adults [30, 31, 32, 33, 34]. Similar to studies of adolescents living with HIV (ALHIV), studies of treatment adherence in adolescents with chronic renal disease find non‐compliance rates in the range of 32% [17] to 38.2% [15], with an average of 11% of doses missed and nearly a quarter of individuals taking medications late [15]. Rates of treatment non‐adherence for adolescent and young adult cancer cohorts range from 27% to 60% [33]. Adolescents who view themselves as different and less worthy than their peers because of their illness are likely to become socially isolated, have a poorer self‐concept, lowered academic functioning and behavioural problems [35]. At the same time, young people who are able to focus on the positive aspects of their condition have more favourable treatment outcomes and recovery from an illness compared to those who do not [32, 36, 37]. The bi‐directional relationship(s) between mental health and chronic disease outcomes, including HIV, necessitate evidence‐based models that address the unique physical and mental health needs of patients during adolescence and emerging adulthood [38, 39, 40, 41].

In addition to clinical determinants (e.g. disease severity, access to treatment), psychosocial factors play a critical role in determining chronic disease outcomes among adolescents. Many young people have difficulty adhering to tailored treatment regimens due to poorly developed abstract thinking, poor ability to plan, an immature ability to imagine future consequences and a concept of themselves as “bulletproof” or not vulnerable [11, 34, 42, 43, 44]. These issues indicate that preventing long‐term disease‐related complications is—on its own—an insufficient motivator for treatment compliance among chronically ill adolescents; developing greater autonomy of disease management habits and cultivating stronger relationships with peers and social networks are also needed to improve clinical and mental health outcomes for this population.

This paper reports findings from an implementation research project evaluating the impact of a recurring, clinic‐based peer support group for young people living with chronic illness in Cape Town, South Africa. Based on extensive evidence that group support offers psychological benefit for young people living with the same chronic condition [44, 45, 46, 47], this pilot study uses a mixed‐methods design to examine the acceptability, feasibility and preliminary impact of a peer support group for youth who are living with a range of chronic illnesses (including HIV) and participating in one group together. Young people managing chronic illnesses other than HIV frequently experience similar stigma, in relation to medication and daily pill taking, and frequent illness episodes or hospitalisations. Preliminary effectiveness evidence of mixed‐condition peer support groups can inform future randomised trials, and the optimal design of adolescent peer support groups in similar settings.

## 2 METHODS

This pilot study used a mixed methods approach to investigate the acceptability, feasibility and preliminary mental health impacts of the *Better Together* programme.

### 2.1 The Better Together programme

The *Better Together* programme [48] provides a weekly facilitated group session open to all adolescents, ages 13–24, who are living with a chronic illness and receiving care at Groote Schuur Hospital (GSH) in Cape Town, South Africa. GSH is a large public health sector hospital where care is provided free to patients through South African governmental health services. Since 2017, *Better Together* has helped adolescents with a range of chronic conditions (including HIV, renal disease and psychiatric conditions) to (1) build social networks that enhance psychosocial support; (2) develop a sense of belonging with peers; (3) create a space where adolescents can share their experience(s) living with and managing chronic illness; and (4) build empathy among ALHIV and other conditions. Groups are facilitated by a volunteer peer mentor who is also living with a chronic condition, and overseen by clinicians from the hospital's Adolescent Clinicians Group.

Volunteer peer mentors approach patients in the GSH waiting room and invite them to participate in the group sessions. Some adolescents attending GSH also participate in disease‐specific support groups at their individual clinics; every adolescent enrolled in care at GSH is invited to join the *Better Together* groups regardless of whether they are also participating in another group.

Group participants are not required to disclose their disease within the groups, but may choose to do so. Confidentiality is discussed at the beginning and end of every session. A peer mentor supervisor (either a psychologist or a social worker) is present to observe all groups and provide additional support to mentors and group participants where necessary. Groups are divided into structured topics related to general adolescent health and development (e.g. sexual and reproductive health, mental health) and less structured “social” groups. Peer mentors are trained to adjust their topics to the age and cognitive maturity of the group members.

### 2.2 Eligibility, enrolment and informed consent

Enrolment and data collection were conducted at GSH from September 2021 through July 2022. Qualitative data collection included in‐depth interviews (IDIs) with *n* = 20 young people who were living with chronic illnesses and who had participated in at least five *Better Together* peer group sessions prior to study enrolment. Quantitative data on resilience, attitudes towards illness, stigma and mental health were ascertained via self‐report from the *n* = 20 individuals who participated in the qualitative interviews and from an additional *n* = 38 adolescent patients at GSH who had not participated in the *Better Together* program. The research team obtained written parental consent and adolescent assent for participants <18 years, and informed consent from those ≥18 years. Participants for whom the team was unable to gain parental consent and/or adolescent assent were excluded. Disclosure of disease status was not required for study enrolment. Participants were informed via the informed consent/assent forms that all of their personal information, including their history of chronic illness(es), would be kept confidential during and after the study.

### 2.3 Quantitative data collection

Self‐reported demographic and clinical data were obtained at study enrolment. Medical history was extracted from electronic medical records and during GSH clinic visits. Additionally, established measures were administered to all study participants at enrolment to quantitatively assess the psychosocial impact(s) of the peer group intervention. Specifically, we administered the Connor‐Davidson Resilience Scale (CD‐RISC 10) [49, 50], Child Attitude Toward Illness Scale (CATIS) [51, 52, 53], Revised Berger scale of disease‐related stigma [54, 55], HIV‐stigma scale for South African adolescents living with HIV [56] and Beck Youth Inventories Second Edition (BYI‐II) [57, 58]. We have previously administered these measures among adolescents and young adults in South Africa, with evidence of good reliability in isiXhosa‐speaking populations [59, 60]. Appendix S1 includes a description of each psychosocial measure.

### 2.4 Qualitative data collection

IDIs were conducted with *n* = 20 adolescents living with chronic illness who were regular attendees at the *Better Together* peer support group. The IDIs explored adolescents’ acceptability and willingness to participate in the programme, as well as their chronic illness narratives and life experiences. Conducting IDIs is consistent with best practice recommendations for sensitive topics; all qualitative procedures followed best practice guidelines [61, 62]. All participants were recruited from hospital‐based peer support groups using convenience sampling. Adolescents were eligible for the IDIs if they met the following inclusion criteria: (1) 13–24 years of age, had attended at least five *Better Together* peer group sessions; (2) aware of their chronic illness as confirmed through self‐report; and (3) currently or previously on treatment for their chronic illness as confirmed through self‐report.

Interviews were conducted using a semi‐structured interview protocol that was adapted from similar qualitative research with adolescents in South Africa. The protocol explored the following aspects of the *Better Together* programme: (1) barriers and facilitators to participation; (2) acceptability of the programme as a chronic illness management strategy; (3) predicted behavioural impacts of the programme, including coping with a chronic illness and medication adherence; and (4) preferred programme format. Interviews were conducted in the participant's language of preference (English or isiXhosa). Interviews lasted approximately 1–1.5 hours and were digitally audiotaped. Interviewees received R250 Rand (∼$15) for their time and transport.

### 2.5 Analytic approach

Quantitative analyses were conducted using StataSE 16 software (Stat Corp.). We first report mean values and standard deviations for continuous characteristics or frequencies and percentages for categorical characteristics overall and by peer group participation. Second, a one‐way multivariate analysis of variance (MANOVA) was used to estimate associations between peer group participation and psychosocial wellbeing (i.e. on total scores for the CD‐RISC 10, CATIS, Revised Berger Scale and on BYI‐II T‐scores). MANOVA is appropriate for evaluating mean differences on multiple dependent variables simultaneously while adjusting for intercorrelations among them [63]. ALHIV‐SS data were excluded from the MANOVA because not all participants were living with HIV. Third, multivariate logistic regression models estimated the odds of being screened positive for depression or anxiety given *Better Together* participation (= 1 if attended ≥5 peer groups prior to study enrolment or = 0 if attended 0 peer groups). Models were adjusted for potential sources of confounding at enrolment, specifically age (in years), sex (male/female), household size, housing type (formal or informal), living status of biological mother (alive/deceased) and participation in one or more government social protection programme(s) (yes/no). Stata's margins command was run after logistic regressions to estimate the predictive probability of being screened positive for depression or anxiety given peer group participation. A cut point of ≥19 for the Beck Youth Depression Inventory (BDI‐Y) raw score was considered a positive screening for moderate to severe depression [64]. A cut point of ≥20 for the Beck Youth Anxiety Inventory (BAI‐Y) raw score was considered a positive anxiety screening [65]. Quantitative results were interpreted using point estimates, confidence intervals (CIs), and *p*‐values; we did not consider an association to be statistically significant based solely on whether *p*‐values were below a given threshold [66, 67]. For all models, alpha was set at 0.10 following literature which suggests that less stringent *p*‐values may be more relevant for identifying meaningful associations in smaller studies [68, 69].

Qualitative data were transcribed verbatim and translated into English as needed. All transcripts were double‐coded. We use a general inductive approach to analyse the qualitative data. Common words, phrases, sentences and ideas were clustered to develop a codebook. Data were analysed using open‐coding, axial coding, and coding of marginal remarks and comparisons [56] using NVivo (QSR International, 2012), followed by thematic analysis of specific codes and sub‐codes. Meaning from these codes was formulated to produce clusters of themes, which were then discussed and agreed on in the coding team.

### 2.6 Ethical approval

All study procedures were approved by the University of Cape Town Human Research Ethics Committee (HREC Approval Number: 075/2020). Written parental consent and written adolescent assent were obtained for participants <18 years of age, and written informed consent was obtained from at least 18 years of age. All human subjects research was conducted by Investigators at GSH. All data that Brown University Investigators received from the University of Cape Town excluded identifiable private information, identifiable biospecimen and coded information, and thus did not meet the definition of human subjects research as defined by *45 CFR 46*.

## 3 RESULTS

### 3.1 Quantitative findings

Fifty‐eight total patients enrolled in the quantitative portion of this pilot study, 20 of whom had attended ≥5 *Better Together* peer support groups prior to enrolment (Table 1). Participants were 18 years of age on average and predominately (71%) Black African. Half (50%) self‐reported their biological sex as female, most (91%) had completed at least some secondary schooling, and 43% were enrolled in a government social programme. On average, participants came from a six‐person household, and 66% and 53% had a biological mother and biological father who was still living, respectively. Peer group participants were older than non‐peer group patients on average (*p* < 0.001). Twelve of the 20 peer group participants and 32 of the 38 control group patients were living with HIV at study enrolment.

**Table 1:** Characteristics of adolescent participants enrolled in the pilot study, overall and by *Better Together* peer group participation

	Total participants	No Better Together group participation	Has attended at ≥5 Better Together group sessions	
	*N* = 58	*n* = 38	*n* = 20	*p*‐value^a^
Age	18.74 (2.84)	17.26 (2.20)	21.55 (1.43)	<0.001
Biological sex^b^				1.00
Female	50% (29)	50% (19)	50% (10)	
Male	48% (28)	47% (18)	50% (10)	
Other	2% (1)	3% (1)	0% (0)	
Race				0.40
Black African	71% (41)	74% (28)	65% (13)	
Coloured^c^	28% (16)	26% (10)	30% (6)	
Indian or Asian	2% (1)	0% (0)	5% (1)	
Language				0.22
IsiXhosa	57% (33)	58% (22)	55% (11)	
Afrikaans	9% (5)	13% (5)	0% (0)	
English	33% (19)	26% (10)	45% (9)	
Other	2% (1)	3% (1)	0% (0)	
Highest level of education completed				0.15
Primary (< grade 8)	9% (5)	13% (5)	0% (0)	
Secondary (≥ grade 8)	91% (53)	87% (33)	100% (20)	
Household size (adults and children)	5.72 (2.39)	6.05 (2.43)	5.10 (2.25)	0.15
Housing type				0.49
Formal	81% (47)	84% (32)	75% (15)	
Informal	19% (11)	16% (6)	25% (5)	
Is biological mother alive?				0.77
No	34% (20)	37% (14)	30% (6)	
Yes	66% (38)	63% (24)	70% (14)	
Is biological father alive?				0.77
No	40% (23)	39% (15)	40% (8)	
Yes	53% (31)	58% (22)	45% (9)	
Unknown	7% (4)	3% (1)	15% (3)	
Currently accessing a government social grant				0.089
No	55% (32)	47% (18)	70% (14)	
Yes	43% (25)	53% (20)	25% (5)	
Missing	2% (1)	0% (0)	5% (1)	

*Note*: Data in Table 1 are presented as mean (SD) for continuous variables and % (*n*) for categorical variables.

^a^ Differences between peer group participants and control patients were assessed using a two sample *t*‐test and a Fisher's exact test for continuous and categorical variables, respectively.

^b^ Survey respondents self‐reported their biological sex as “Male,” “Female” or “Other” in response to “Would you define your sex as?”.

^c^ Coloured is an official, socially acceptable racial classification defined by South Africa's Population Registration Act of 1950.

Table 2 shows the average total score on each psychosocial measure for the two study groups. Compared to youth patients who did not participate in any *Better Together* peer groups, those who had attended at least five peer group sessions had higher self‐reported individual‐level resilience (*p* = 0.004), a more positive attitude towards their chronic illness(es) (*p* < 0.001), lower internalised stigma related to their chronic disease (*p* = 0.006), stronger self‐concept (e.g. confidence in self, positive body image, relatability to others) (*p* = 0.039) and lower depressive symptoms (*p* < 0.10). At α = 0.10, average total or T‐scores on the ALHIV‐SS HIV stigma scale, Beck Anxiety Inventory for Youth, Beck Anger Inventory for Youth and Beck Disruptive Inventory for Youth did not statistically significantly differ between peer group and non‐peer group patients. Results of the one‐way MANOVA confirmed the aforementioned associations, showing a statistically significant association between mental health improvements as a function of peer group participation overall, (Wilks’ Lambda = 0.7349, *F* (8,49) = 2.21, *p* = 0.043), and specifically for the individual domains of resilience, attitude towards illness, chronic disease stigma, self‐concept and depression.

**Table 2:** Mean total scores on psychosocial measures among adolescent patients with and without exposure to the *Better Together* peer group intervention

	Summary score interpretation	Non‐peer group patients (Control group)	Peer group participants (Intervention arm)	*p*‐value^a^
10‐item Connor‐Davidson Resilience Scale (CDRISC‐10)	Higher score indicates greater individual resilience	26.47 (6.79)	31.40 (4.03)	0.004
13‐item Child Attitude Towards Illness Scale (CATIS)	Higher score indicates a more positive attitude towards chronic illness	45.37 (8.52)	53.50 (6.92)	<0.001
Revised Berger Scale of Chronic Disease‐Related Stigma	Higher score indicates more frequent chronic disease‐related stigma	37.84 (7.73)	31.75 (7.61)	0.006
10‐item HIV Stigma Scale for South African Adolescents Living with HIV (ALHIV‐SS)	Higher score indicates more frequent HIV‐related stigma	7.31 (3.35)	6.17 (2.12)	0.28
Beck Self‐Concept Inventory for Youth	Higher score indicates elevated (more positive) self‐concept symptoms	51.53 (11.16)	57.20 (5.93)	0.039
Beck Anxiety Inventory for Youth	Higher score indicates elevated anxiety symptoms	56.53 (12.91)	53.60 (9.58)	0.38
Beck Depression Inventory for Youth	Higher score indicates elevated depressive symptoms	52.37 (10.78)	47.65 (5.26)	0.071
Beck Anger Inventory for Youth	Higher score indicates elevated anger symptoms	51.29 (9.75)	48.85 (4.79)	0.30
Beck Disruptive Inventory for Youth	Higher score indicates elevated disruptive symptoms	49.34 (8.05)	47.70 (5.21)	0.41

*Note*: Scores are presented as mean scores with standard deviations in parentheses.

^a^ Differences in mean measure scores between peer group participants and control patients were assessed using a two sample *t*‐test.

For all measures other than the ALHIV‐SS, *n* = 58 (20 peer group participants and 38 control group patients). For the ALHIV‐SS, *n* = 44 (12 peer group participants and 32 control group patients).

Patients were required to have attended ≥ 5 peer groups at study enrolment to be included in the treatment arm. Control patients had never participated in the *Better Together* peer group intervention.

Scores for the Becky Youth Inventories (BYI‐II) represent T‐scores assigned by age and gender. On each of the BYI‐II subscales, a T‐score of ≤55 = average symptoms, 55–59 = mildly elevated symptoms, 60–69 = moderately elevated symptoms and ≥70 = extremely elevated symptoms.

Table 3 presents the results of logistic regression models estimating associations between peer group participation and depression or anxiety. In all models, attending ≥5 group sessions was positively associated with a reduced odds of being screened positive for depression or anxiety. In adjusted models, relative to adolescent patients who did not participate in peer groups, the odds of being screened positive for moderate to severe depression was 0.08 times lower among peer group participants (95% CI: 0.00, 1.50), and the odds of being screened positive for anxiety was 0.053 lower among peer group participants (95% CI: 0.01, 0.57). The predicted probability of being screened positive for moderate to severe depression was 23.4 percentage points lower for young adults participating in the *Better Together* programme compared to non‐peer group patients (95% CI: 1.5, 45.3; *p* = 0.036); the probability of a positive anxiety screening was 45.8 percentage points lower among peer group participants (95% CI: 18.1, 73.6; *p* = 0.001).

**Table 3:** Results of logistic regression analyses estimating associations between participation in peer support groups and being screened positive for depression or anxiety (*N* = 58 adolescents living with chronic illness)

		(1)	(2)	(3)	(4)
		Positive depression screening	Positive depression screening	Positive anxiety screening	Positive anxiety screening
		OR (95% CI)	aOR (95% CI)	OR (95% CI)	aOR (95% CI)
Participation in ≥ 5 peer group sessions	No	(ref)	(ref)	(ref)	(ref)
	Yes	0.13^*^	0.08^*^	0.38	0.05^**^
		(0.02, 1.09)	(0.00, 1.50)	(0.11, 1.37)	(0.01, 0.57)
Age in years			1.04		1.41^*^
			(0.70, 154)		(0.96, 2.07)
Biological sex	Female		(ref)		(ref)
	Male		0.60		0.30^*^
			(0.12, 2.94)		(0.07, 1.25)
Household size (adults and children)			1.00		1.00
			(0.69, 1.41)		(0.72, 1.38)
Housing type	Formal		(ref)		(ref)
	Informal		0.41		0.43
			(0.03, 5.16)		(0.06, 3.19)
Status of biological mother	Deceased		(ref)		(ref)
	Alive		0.18^**^		0.41
			(0.03, 0.91)		(0.09, 1.80)
Participating in government social protection programme	No		(ref)		(ref)
	Yes		0.36		0.21^**^
			(0.08, 1.79)		(0.05, 0.97)

*Note*: A cut point of ≥ 19 on the Beck Youth Depression Inventory (BDI‐Y) raw score was considered indicative of a positive depression screening [64]. A cut point of ≥ 20 on the Beck Youth Anxiety Inventory (BAI‐Y) raw score was considered indicative of a positive anxiety screening [65].

Abbreviations: aOR, adjusted odds ratio; CI, confidence interval; OR, odds ratio.

*p < 0.10; **p < 0.05; ***p < 0.010.

### 3.2 Qualitative findings

Across interviews, all participants emphasised the value of being able to compare treatment regimens and personal stories with peers living with different conditions. ALHIV stated frequently that understanding the hardships faced by those with other conditions helped with acceptance of their own illness and lessened feelings of isolation. Similarly, youth living with other chronic conditions experienced increased social support and reduced isolation. Although disclosure was not required within the groups, many participants described the process of becoming comfortable discussing their illness with peers.

*Theme 1 Meeting young people with different illnesses was an eye‐opening and powerful experience for most young people*.

The participants found support from youth living with different illnesses, and learned about the common challenges faced by people with HIV, diabetes or chronic conditions, such as kidney disease. When asked about attending the peer support group with other youth living with different illnesses, one participant responded that the experience helped to combat the feeling of isolation by connecting around challenging aspects of living with a chronic illness:

That was a very powerful move. I don't know who thought about it, but it was so powerful. We started meeting with other groups. We are told that we're going to say what we really have. And when we got the group, we started looking at each other there and there was a bit of fear. And then I said, guys, ‘I'm… thought I should put it out there guys. I'm positive.’ And then the next fellow… And then other people asked, how does it feel to be positive? And then we got information upon information. (PID00009; Male aged 24, living with HIV)

The same participant continued:

I remember one person who said I wish I was positive, and then I asked why …? And then they said having a kidney failure is the worst. … then they mentioned that they take 21 tablets a day; by saying that the whole group said, Wow, 21 tablets. And then they asked how many tablets do you guys take? Some people said three tablets, you only take three tablets and there are people who find it hard to take three tablets. That kind of made me feel that maybe I'm not having the worst. So I am just overreacting, let me take my medication. (PID00009; Male age 24, living with HIV)

Another participant agreed, comparing his concerns with adherence to HIV medication as easy, compared to a diabetes patient, “someone who pricks him or herself every hour.” Others reflected on the benefits of meeting peers with different chronic illnesses:

To be honest, I am enjoying much more than the group I had whereby it's just people with my chronic illness. I mean, it is nice to have a group of people with the same illness, but to me I feel like it is nice to get to chat with people with different illnesses because you hear different experiences from them and even though we may think that it is different, but we have many similarities and things that we go through. (PID 00001, Male age 20, living with renal disease)

Overall, youth were positive about their participation in the peer support groups, and saw added benefits from meeting and interacting with other young people, sharing the common experience of living with chronic illness and the challenges this poses for a young person, including frequent feelings of isolation.

*Theme 2 Young people living with chronic illness frequently encounter stigma and uninformed attitudes in school and the community—they reported finding support and acceptance in the support group, among peers living with diverse chronic illnesses*.

One participant discussed learning about her HIV condition at age 11, after she inquired about why she was taking pills, only to learn that her father had different partners and infected her mom. She described her personal journey of adjusting to a future with a chronic illness, in this case, HIV.

I live with this thing that cannot be cured. … But I learned to accept it because I thought to myself, you know what? I can look at this from a different point of view. I thought about so many people who are out there successful, who are HIV positive. Because it does not mean a death sentence when you are HIV positive. (PID 00015; Female Age 22, living with HIV)

This participant also spoke about finding it easier to accept her status by seeing how others navigate their chronic illnesses:

If we were to look at all sicknesses, I would rather have HIV. For example, with diabetes, some people have to get rid of some of their body parts. There's a lot of sicknesses that are unbearable. There are even diseases that affect your lungs, so I'd rather be HIV positive because being HIV positive, you can look at me, you won't see it. You will see a beautiful round girl… (PID 00015; Female Age 22, living with HIV)

A common theme among participants living with chronic illnesses was that participation in groups allowed them to consider the future, and the unique challenges of navigating futures—life goals, marriage and children—and to see the future as a possibility. For example, the following adolescent who required a kidney transplant described how the group supported a view of the future being a real possibility:

I won't lie … it does affect me because I am human, there are some days where I would feel like if I did not have this, things would have been much better. But now I am very much in acceptance. My life has gotten easier with the transplant. I don't feel as weak as I used to. Things are getting better and the part of people telling me that my having this and still pursuing my dreams is inspiring them. And I bring birth to new dreamers, and people who want to achieve goals. (PID 00001, Male Age 20, living with kidney failure/renal disease)

Another participant spoke about the challenges of living with cancer while also raising a young child:

I am grateful for the things that I have achieved. I feel like there have been so many blessings that came my way, so much mercy has been shown up on me and even my daughter is a miracle. OK, but circumstances of how it happened were not what I wanted, of course, because I was in my matric year, but because of the radiation that I went for my spinal operation it was said that I wouldn't be able to have children. And then somehow, I have my daughter, so she is my miracle. (PID 00002; Female Age 21, living with cancer)

Across illness types and experiences, youth in the peer support groups found a safe place to express themselves freely, and to be understood. Additionally, many youth faced challenges at home in addition to the already heavy burden of living with a chronic illness as a young person. Some participants mentioned additional and unexpected benefits they derived from the need to take care of themselves, such as being removed from the culture of drugs and alcohol use that is prevalent among youth in many communities. Further, they learned specific skills to manage their own illness and tools to address the mental health consequences of being a young person living with a serious illness.

*Theme 3 Young people with a variety of chronic illnesses described the benefits of participation in the peer support group*.

Young people reflected on the vital importance of social support provided by the peer mentor and peer support program. Below, an interviewer (I) and a peer group participant (P) discussed this:

I: Now you have highlighted that it feels like a family away from your family. You have different chronic illnesses, but you see similarities and you take yourself as just one….

P: I know I would get more and more support. I know that I would probably know things that I did not know before. There are so many different people, so I get to experience different personalities. …. And how it has helped me is as I said, everyone has a different illness. And as you have mentioned that sometimes chronic illnesses are stigmatised in societies, so we don't always exactly know what it means or what it's about, how it works, or how to interact with this person. You need to interact in a certain way. So, it teaches me a lot about other illnesses and how to approach or interact with others. (PID 00013; Female Age 22, living with HIV)

The interviewers also asked peer group participants to reflect on anything that was not positive about the groups:

At first, I thought what if they're going to tell someone else? To disclose my status to someone else, and I wasn't ready about it. And I faced that. And then we had this say that says what happens in Vegas stays in Vegas. And then we spoke about it. And then we shared all of our problems, personal problems, and when you leave that door, we said nothing that we say here is going to leave the place. So it was safe for people to actually open up. (PID00009; Male age 24, living with HIV)

Further, dealing with other young people's illnesses is not always straightforward or emotionally easy. Although interacting with other young people with chronic illness was perceived as widely beneficial, there is an emotional toll from such participation, including sharing one's story and working to understand and provide support to others. One participant described coming to terms with their own illness:

I have accepted it. No one is motivating me more than the chronic illness itself; I've learned that time management is important. Secondly, determination is also important. Everything I do must be positive. I am proud about the fact that I am HIV positive. So, I have to be positive in life and see light at the end of the tunnel every time. (PID 00008, Male Age 22, living with HIV)

Finally, one of the peer mentors explained their role:

For me it is an honor because I get to understand other patients from other clinics, I get to actually understand their illness, the things they go through. For me it is an honor in a way that I also get to meet new people because I actually meet new people. I did not know people I can have conversation with. I get to experience some new people or get some new information from them. (PID 00003, Age 20, living with HIV)

## 4 DISCUSSION

This mixed methods analysis of pilot data from a clinic‐based peer support group intervention in Cape Town, South Africa found encouraging psychosocial and mental health benefits of providing peer support groups to adolescents living with diverse chronic conditions. Quantitative analysis shows several benefits associated with the *Better Together* programme, with participants of five or more sessions exhibiting better outcomes across a number of psychosocial measures. The qualitative data are highly complementary, with detailed illness narratives giving insights into young people's need for enhanced social support in relation to living with chronic illness, and their reflections on how the peer support programme helped them to meet those needs. Patients who attended five or more *Better Together* peer support group sessions exhibited enhanced individual resilience, greater acceptance of their chronic illness, lower internalised stigma, a stronger self‐concept and lower depressive symptoms. In multivariable logistic regression models, attending at least five group sessions was statistically significantly associated with a reduced odds of screening positive for anxiety (*p* < 0.05) or moderate to severe depression (*p* < 0.10), compared to non‐peer group patients. This corresponded to a 23%–45% reduction in the probability of a positive depression or anxiety screening given peer group participation.

The interview data underscore some of the potential reasons for these positive outcomes, and provide insight into young people's quality of life related to living with chronic illnesses, including HIV. Irrespective of diagnosis, young people described facing stigma and unwanted attention related to their condition and frequent social isolation due to living a life that is distinctly different from their non‐chronically ill peers. Participants voiced that a key benefit of the peer support group was the space it gave them to interact with and learn from peers who are living with other conditions but still facing similar disease management challenges. The qualitative data also provide insight into the specific benefits of a mixed chronic illness group for youth living with HIV. The experience of engaging with other adolescents who have different chronic illnesses seemed to normalise HIV as a chronic illness “just like any other illness.” Many adolescents with HIV were able to reflect on the burden of managing HIV as being something that seems more manageable than diabetes or cancer. Learning about other chronic illnesses seemed to mitigate some of the internalised stigma of living with HIV which is a persistent barrier to treatment adherence and care engagement.

This mixed methods analysis builds on our prior work [70, 71] and adds to the body of evidence around peer support programs for adolescents with chronic illnesses. Peer support models are recommended as part of best practices to improve youth medication adherence [39, 72], reduce disease‐related stigma [73, 74] and improve other mental health outcomes [38]. Yet, to the best of our knowledge, the current effectiveness evidence of these models is overwhelmingly derived from approaches that provide peer support to youth living with the same chronic condition [73, 74, 75, 76, 77]. Focusing solely on one chronic condition may limit the full potential of peer support groups by preventing youth from capitalizing on the increased knowledge that stems from sharing experiences and disease management habits across conditions. The present analysis advances the evidence [78, 79] by demonstrating that routine peer support groups for youth with diverse chronic conditions can expand an individual's disease management toolkit, and increase their ability to empathise with others living with unique disease‐related challenges.

### 4.1 Limitations

A main limitation of this preliminary efficacy assessment is the small sample size; qualitative data were ascertained from 20 interviews, while quantitative survey data were ascertained from 58 adolescent patients of the hospital. Thus, our findings of positive associations between peer group participation and better psychosocial health should be interpreted with caution, in light of the modest sample size and potential for sampling and selection biases. Further research is needed to assess the impact in a larger sample over time. Also, the study site comprises one location, in one particular setting, and it is possible that different youth populations might require a different approach.

## 5 CONCLUSIONS

This pilot evidence supports the acceptability, feasibility and potential positive mental health impact(s) of a peer support group that includes young people living with diverse chronic diseases. The acceptability of this approach is high, with young people expanding on multiple benefits that they receive from interacting with other youth living with varied chronic illnesses. Feasibility has been well demonstrated with the continuation of the *Better Together* programme since 2017, including during disruptions of the COVID‐19 pandemic in 2020 and 2021. To fully understand the effectiveness and causal mechanisms of mixed condition peer groups on mental and social health outcomes, a fully powered randomised trial is needed.

## COMPETING INTERESTS

All authors assert no competing interests.

## AUTHORS’ CONTRIBUTIONS

AH, OG, BD, RS and JH led the conception and design of the work. BM, BD and RS were responsible for the acquisition and interpretation of the data. AH, MW‐B, CK and OG led data analysis. AH and MW‐B wrote the first draft of the manuscript. All authors revised the work critically for important intellectual content, and agree to be accountable for all aspects of the work in ensuring that questions related to the accuracy or integrity of any part of the work are appropriately investigated and resolved.

## FUNDING

Research reported in this publication was supported by funding from One to One Children's Fund. We acknowledge the Population Studies and Training Center (PSTC) at Brown University, which receives funding from the NIH (P2C HD041020), for general support. [Correction Added on 11 January 2024, after first online publication: Author Contributions, Acknowledgement and Funding Sections have been modified.]

## Supporting information


**Appendix 1**: Summary of psycho‐social measures administered to N = 58 study participants.

## Data Availability

Following publication, the de‐identified data and analytic code that support the quantitative findings in this manuscript are openly available in the Brown University Digital Repository at https://doi.org/10.7910/DVN/NSCUS4.
