# Correction to “Better together: acceptability of chronic illness peer support groups for South African adolescents and young adults”

**DOI:** 10.1002/jia2.26209

**Published:** 2024-01-28

Harrison A, Mtukushe B, Kuo C, Wilson‐Barthes M, Davidson B, Sher R, et al. Better together: acceptability of chronic illness peer support groups for South African adolescents and young adults. *J Int AIDS Soc*. 2023;26(S4):e26148. https://doi.org/10.1002/jia2.26148

In the article, the Authors’ Contributions, Acknowledgements and Funding sections are wrong.

The incorrect sections are:

## AUTHORS’ CONTRIBUTIONS

AH, OG and JH led the conception and design of the work. BM, BD and RS were responsible for the acquisition and interpretation of the data. AH, MW‐B, CK and OG led data analysis. AH and MW‐B wrote the first draft of the manuscript. All authors revised the work critically for important intellectual content, and agree to be accountable for all aspects of the work in ensuring that questions related to the accuracy or integrity of any part of the work are appropriately investigated and resolved.

## FUNDING

Research reported in this publication was supported by private funds obtained by South African investigators (JH, BD and RS). We acknowledge the Population Studies and Training Center (PSTC) at Brown University, which receives funding from the NIH (P2C HD041020), for general support.

The correct sections are:

## AUTHORS’ CONTRIBUTIONS

AH, OG, BD, RS and JH led conception and design of the work. BM, BD and RS were responsible for the acquisition and interpretation of the data. AH, MWB, CK and OG led data analysis. AH and MW‐B wrote the first draft of the manuscript. All authors revised the work critically for important intellectual content, and agree to be accountable for all aspects of the work in ensuring that questions related to the accuracy or integrity of any part of the work are appropriately investigated and resolved.

## FUNDING

Research reported in this publication was supported by funding from One to One Children's Fund. We acknowledge the Population Studies and Training Center (PSTC) at Brown University, which receives funding from the NIH (P2C HD041020), for general support.

The online version of the article has been corrected.

