# Supplementary material for: Better Together: acceptability, feasibility and preliminary impact of chronic illness peer support groups for South African adolescents and young adults
Source: J Int AIDS Soc. 2023 Nov 1;26(Suppl 4):e26148. doi: 10.1002/jia2.26148 (PMC10618872; doi:10.1002/jia2.26148)
Supplement: Supplementary file 1 — Appendix 1: Summary of psycho‐social measures administered to N = 58 study participants. [file JIA2-26-e26148-s001.docx]

**Appendix 1. Summary of psycho-social measures administered to n=58 study participants**

| **Measure** | **Description** | **Scoring Approach** | **Score Interpretation** |
| --- | --- | --- | --- |
| **10-item Connor Davidson Resilience Scale (CDRISC-10)** | 10-item scale capturing individual-level resilience over the last month (e.g., adaptability to change, goal achievement, facing obstacles); response options range from 0 (not true at all) to 4 (true nearly all of the time). | Total scores range from 0 – 40 | Higher score indicates greater individual-level resilience |
| **13-item Child Attitude Towards Illness Scale (CATIS)** | 13-item scale measuring children’s self-reported attitudes towards their chronic condition(s); response options are on a 5-point scale using opposing adjectives (e.g., very good (1) to very bad (5), very sad (1) to very happy (5)). | A summary score is generated by reverse scoring scale items 1, 2, 4, 5, 7, 9, 11, and 13, and then summing all items and dividing by 13.  Summary scores range from 13 to 65 | Higher score indicates a more positive attitude towards chronic illness |
| **Revised Berger Scale of Chronic Disease-Related Stigma** | 14 items adapted from the validated 40-item Berger HIV stigma scale that assess self-reported stigma experienced due to living with one or more chronic conditions (including but not limited to HIV). Response options for each item range from 1 (strongly disagree) to 5 (strongly agree). | Scores are summed to generate a total score ranging from 14 to 70 | Higher score indicates more frequent chronic disease-related stigma |
| **10-item HIV Stigma Scale for South African Adolescents Living with HIV (ALHIV-SS)** | 10-item scale assessing self-reported HIV-related stigma across three domains: internalized, anticipated and enacted stigma. Response options are on a 3-point Likert scale (0: Never; 1: Sometimes; 2: Always) | Scores are summed to get a total score ranging from 0 to 20 | Higher score indicates more frequent HIV-related stigma |
| **Beck Youth Inventories Second Edition (BYI-II)** | Inventories are structured in line with DSM-IV-TR criteria and used to assess symptoms of depression (BDI-Y), anxiety (BAI-Y), anger (BANI-Y), disruptive behaviour (BDBI-Y), and self-concept (BSCI-Y) among young people age 7-18 years.  For each of the five inventories, both a raw total score and a T-score assigned by age and gender are generated. | For each inventory, a T-score of <55 = average symptoms; 55-59 = mildly elevated symptoms; 60-69 = moderately elevated symptoms; and >70 = extremely elevated symptoms. | Higher score indicates elevated anxiety symptoms, elevated depression symptoms, elevated anger symptoms, elevated disruptive symptoms, or elevated (more positive) self-concept symptoms |
| DSM-IV-TR: Diagnostic and Statistical Manual of Mental Disorders, 4th Edition, Text Revision | | | |
